# Investigating Cross-Linking Parameters and Molecular Arrangement in Liquid Crystalline Epoxy Monomer with Aromatic Diamine: DSC-TOPEM^®^ and WAXS Analysis

**DOI:** 10.3390/polym16142034

**Published:** 2024-07-17

**Authors:** Weronika Zając, Beata Mossety-Leszczak, Maciej Kisiel, Magdalena Włodarska, Piotr Szałański

**Affiliations:** 1 Doctoral School of the Rzeszow University of Technology, al. Powstańców Warszawy 12, 35-959 Rzeszow, Poland; 2 Department of Industrial and Materials Chemistry, Rzeszow University of Technology, al. Powstańców Warszawy 6, 35-959 Rzeszow, Poland; m.kisiel@prz.edu.pl; 3 Institute of Physics, Lodz University of Technology, Wólczańska 217/221, 93-005 Lodz, Poland; magdaw@p.lodz.pl; 4 Laboratory of Spectrometry, Rzeszow University of Technology, al. Powstańców Warszawy 12, 35-959 Rzeszow, Poland; pszalans@prz.edu.pl

**Keywords:** cross-linking, nanocomposite, crystal structure, differential scanning calorimetry, epoxy matrix, WAXS

## Abstract

This study presents the characterization of cross-linking parameters of a liquid crystalline epoxy monomer with an aromatic diamine as a curing agent. The mixture tested consisted of bis [4-(10,11-epoxyundecanoyloxy)benzoate] p-phenylene (LCEM) and 1,3-phenylenediamine (1,3-PDA). This paper focuses on the structural characterization of such systems using X-ray analysis. To investigate this, a comprehensive analysis was conducted using Differential Scanning Calorimetry (DSC) and Wide-Angle X-ray Scattering (WAXS). Through DSC analysis, the curing behavior and transition temperature of the liquid crystal epoxy system were established. To fully characterize the cross-linking of the system, a novel technique called DSC-TOPEM^®^ was employed. The use of this technique enabled real-time monitoring of the curing process and provided precise information on the cross-linking energy, which resulted in the finding that the mixture was cross-linking faster than expected. WAXS analysis was performed to assess the structural changes formed during the cross-linking. The results of this analysis confirm that lower cross-linking temperatures of the mixture cause better ordering of mesogens than higher ones.

## 1. Introduction

Liquid crystals have gained significant importance in various industries and applications. Their unique properties make them valuable in fields such as electronics, optics, materials science, and biotechnology. Research that includes such compounds is a response to the growing demand for materials with specific properties, such as chemical resistance, adhesion to various surfaces, or rigidity [1,2]. These properties can be achieved through the synthesis of liquid crystalline epoxy monomers (LCEM). Such materials, after curing, are characterized by a high glass transition temperature, better stability, and mechanical properties [3,4]. They owe this to mesogenic groups, that is, structural elements typical of liquid crystals. Mesogens are parts of the molecular structure and are responsible for shaping the mesophase of thermotropic liquid crystals in appropriate temperature ranges. These rigid elements often consist of several aromatic rings (usually 2–4) linked in diverse ways, with various aliphatic chains attached as side branches. However, these chains must contain functional groups capable of cross-linking [2]. There are multiple ways of joining aromatic rings that can be found in the literature. Generally, the most popular are ester or ether bonds due to the relatively easy method of synthesis of these compounds and the limited flexibility of such bonds [5,6]. There are also other types of linkage, such as the azo bond, which is the most common chromophore introduced into the polymer system. Its presence allows the compound to be used as part of optical memory, sensor construction, and molecular switches [7,8]. The use of an aliphatic chain of various lengths in the mesogenic part of the compound is also practiced.

The mesogenic core significantly influences the behavior of the entire system during the cross-linking process with hardeners, with and without the presence of fillers. In turn, the length of the side chain in liquid crystal molecules can significantly affect the flexibility of the system after curing. Also, the type of bonds between carbon atoms in the chains can affect flexibility; carbon-carbon single bonds are more flexible than double or triple bonds. This is particularly evident when used in fatty acids synthesis [9].

Advanced, liquid crystalline polymer composites in which the basic element was a matrix consisting of LCEM and various additives were also synthesized. The key process in the synthesis of liquid crystal epoxy composites is the curing of the system. The material can be cross-linked by epoxy photopolymerization, and in the most common way—chemically—using a suitable curing agent [10]. In terms of the selection of the cross-linking agent, liquid crystals are similar to ordinary epoxy compounds. There are many types of compounds that can fulfill this function. Acid anhydrides and amines are the most popular compounds among them. Anhydrides are widely available and cheap, but their use requires high temperatures for curing reactions [11].

Amines do not require a significant increase in the system temperature during the cross-linking process. Even with aromatic amines being very popular in the literature, curing can be performed below 100 °C [11]. The presence of functional groups at different positions in relation to the aromatic ring in the amine and the amount of these groups affect the ability of the amine to form polymer networks. Most often, primary or secondary amines are used because of the easy possibility of combining them in a stoichiometric ratio with the epoxide monomer, so that there is one epoxide group for one hydrogen atom of the amino group. One of the most common hardeners of this type is 4,4′-diaminophenylmethane (4,4′-DDM), p-aminophenyl sulfone (p-DDS), and phenylenediamine (PDA) with different isomeric positions of the substituent. Epoxy compounds cured with 1,3-phenylenediamine have high heat resistance, good chemical resistance, and dielectric properties [12].

In their previous research, the authors demonstrated that it is possible to control the alignment of elongated nanofiller particles within a matrix containing mesogenic diepoxide molecules by applying a strong magnetic field. The influence of various amine curing agents on the behavior of the mixture under similar conditions was also observed. They continued their studies to thoroughly analyze the effects of the magnetic field and the interactions between the matrix and nanofiller on various aspects, including morphology, the degree of overall alignment, and thermo-mechanical properties [10,13,14,15,16].

In the presented paper, we describe the study of the influence of the magnetic field on the ordering of mesogenic groups achieved in a system consisting of a liquid crystal monomer containing a three-ring mesogenic core and one of the isomers of aromatic phenylenediamine as a cross-linking agent. The composition of bis [4-(10,11-epoxyundecanoyloxy)benzoate] p-phenylene (LCEM) and 1,3-phenylenediamine (1,3-PDA) is a novel system and has not been described so far in the literature. This system was subjected to detailed analyses to determine which conditions are the most optimal for obtaining ordered mesogenic structures of the material.

The paper also presents one of the temperature-modulated DSC techniques, called DSC-TOPEM^®^. The key advantage of this advanced technique, introduced by Mettler Toledo, is its ability to separate overlapping thermal events that are not easily distinguishable in conventional DSC. Different thermal transitions in the sample, such as glass transitions, crystallization, melting, and chemical reactions, can be detected and characterized more accurately by applying temperature modulation. The technique provides enhanced resolution and sensitivity, making it particularly useful for studying complex or multi-component materials allowing for the separation of reversing and non-reversing heat flows [17,18]. It is a way of conducting the analysis, which helps in understanding a broader understanding of the thermal process of curing the substance.

WAXS (Wide Angle X-ray Scattering) is a technique widely used in materials science to study the atomic and molecular structures of a wide range of materials [15,16]. In our study, the WAXS analysis was used to determine the distribution of mesogenic molecules in the LCEM/1,3-PDA composition after curing.

## 2. Materials and Methods

**Figure 1:**
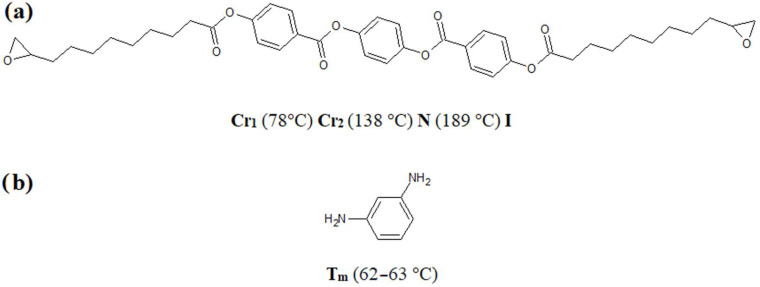
Chemical structures of the diepoxy monomer, the curing agent, and their phase transition data: (**a**) bis [4-(10,11-epoxy undecanoyloxy)benzoate] p-phenylene (LCEM), (**b**) 1,3-phenylene¬diamine (1,3-PDA); Cr_1_—crystalline form 1; Cr_2_—crystalline form 2; N—nematic phase; I—isotropic phase, T_m_—melting point (given temperatures are maximum/minimum peaks) [14,19].

The subject of the research is a liquid crystalline epoxy monomer named bis [4-(10,11-epoxyundecanoyloxy)benzoate] p-phenylene (LCEM), which includes a mesogenic core of three aromatic rings in its structure. It was synthesized according to the procedure described in the article [13]. As a curing agent, 1,3-phenylenediamine (1,3-PDA) was used. All of the reagents were purchased from Sigma-Aldrich, Missouri, USA (products catalog numbers: 8.20991—1,3-PDA; 8.22251—acetone) and used as received without further purification. The structures of the chemical molecules are shown in Figure 1.

### 2.1. Preparation of the Reacting Mixture

**Figure 2:**
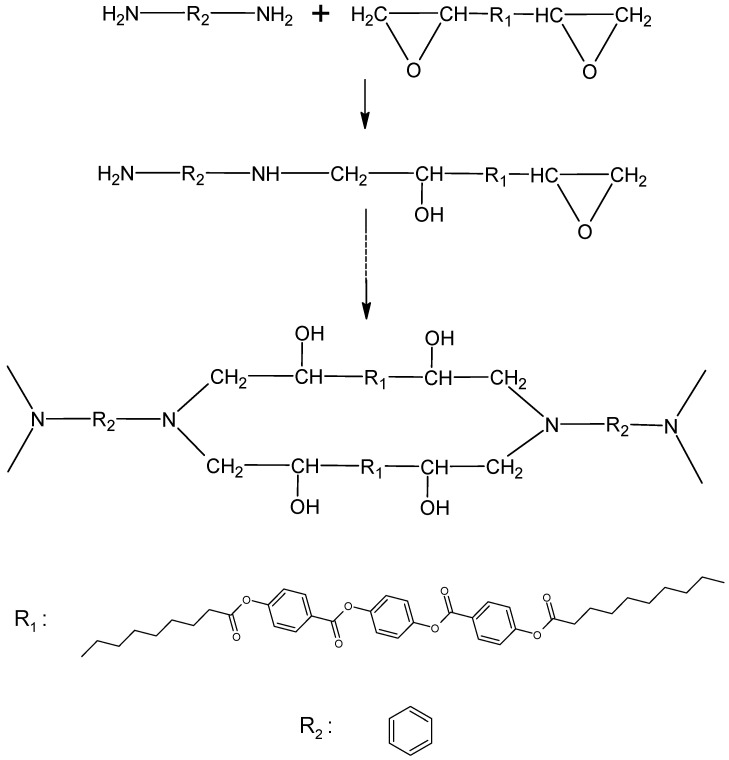
Polyaddition reaction of LCEM with 1,3-PDA.

LCEM was cured in a polyaddition reaction with 1,3-PDA. The molar ratio of the mesogenic monomer to the curing agent was 1:2. The samples of the LCEM/1,3-PDA composition were prepared by suspending the appropriate amounts of LCEM and 1,3-PDA in acetone. The homogeneous sample was then subjected to intense shaking for 3 h. Subsequently, the solvent was evaporated under a vacuum at room temperature and the powdered product was stored at 5–10 °C. The schematic representation of this reaction is shown in Figure 2.

### 2.2. DSC Analysis

Differential scanning calorimetry analysis was used as the main analytical technique to establish adequate curing conditions. The DSC analyses were performed on a Mettler Toledo DSC822e instrument with STARe System software 16.20. Samples were prepared in aluminum crucibles with a capacity of 40 microliters. The analyses were carried out in dynamic temperature increase mode and under isothermal conditions, both in the presence of a nitrogen gas atmosphere. The calibration of the apparatus was made with indium and zinc supplied by Mettler Toledo. First, the pristine components of the mixture were analyzed separately. Samples of both the liquid crystalline epoxy monomer LCEM and the diamine were heated from 0 to 280 °C at a heating rate of 5 °C min^–1^ to determine their phase transition temperature. Analogous tests were performed for the LCEM/1,3-PDA mixture. That sample was also overheated twice in a range of 0–280 °C at a heating rate of 10 °C min^−1^ to observe whether the sample was fully cured and to determine the glass transition temperature (T_g_). The T_g_ of the sample was determined based on the standard PN-EN ISO 11357-2:2020-09 [20].

Subsequently, the examinations of the LCEM/1,3-PDA mixture were continued under isothermal conditions. Samples were prepared in two ways. One part of them was kept for 3 h under constant temperature conditions, and the other part was cured in two steps: First, for 3 h at the constant temperature, followed by post-curing for 1 h at a higher temperature. Then they were overheated in the same way as in dynamic analyses before.

#### Temperature-Modulated DSC TOPEM^®^

Temperature-modulated differential scanning calorimetry (TM-DSC) is a specialized technique used to analyze the thermal properties of materials. It is an advanced variation of differential scanning calorimetry (DSC) that provides additional information about the sample beyond what traditional DSC can offer [17]. This paper used the DSC-TOPEM^®^ technique as a variant of the TM-DSC. DSC analyses with TOPEM^®^ temperature modulation were carried out under nitrogen with a flow of 60 mL min^−1^, at a heating rate of 2 °C min^−1^, with a pulse height of 1 K (±0.500 K) and pulse width of 15–30 s, within a temperature range of 0–270 °C.

### 2.3. Curing

**Figure 3:**
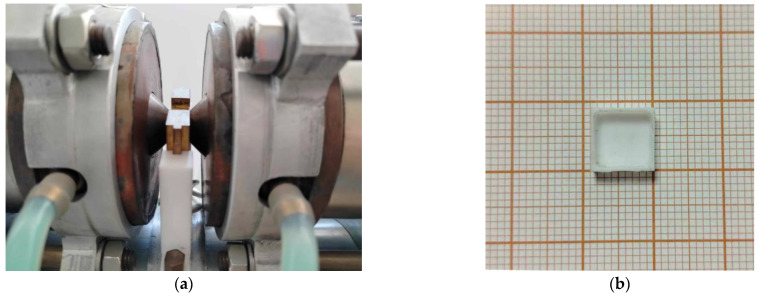
(**a**) Device for generating a uniform magnetic field RTM-1 and (**b**) Teflon mold used during curing.

Thanks to DSC analysis, the most optimal conditions for cross-linking of the liquid crystalline epoxy composition LCEM/1,3-PDA were established. Samples of the mixtures were placed in Teflon molds (7 mm × 3 mm × 7 mm) and cured under the conditions specified in Table 1. Isothermal conditions were determined based on the temperatures at which the cross-linking process begins. The isothermal analyses conducted at specific temperatures are presented in Section 3.1.2. The samples were cured in a magnetic field using an RTM-1 device from REMEL S.C., Nowy Targ, Poland, equipped with neodymium magnets capable of providing a homogeneous magnetic field of induction up to 1.2 T (Figure 3).

During the process, the LCEM/1,3-PDA composition was heated in the presence of a magnetic field applied to the heating device. The lines of the magnetic field were parallel to one of the longest edges of the samples. Subsequently, the samples were cooled to room temperature. For a better interpretation of results, analyses were carried out without any magnetic field, under the same temperature conditions.

### 2.4. WAXS Analysis

Wide-angle X-ray diffraction analyses (WAXS) for cross-linked samples were conducted using a NanoStar-U diffractometer (Bruker, Billerica, MA, USA) with a two-dimensional detector in a transmission configuration. X-rays with a wavelength of approximately 1.54 angstroms (Å) were generated by a copper lamp radiating at 600 mA and 50 kV. The diffractometer was equipped with two Göbel mirrors to monochromate and align the 500 mm diameter beam in a parallel manner. The measurements were carried out under normal room temperature conditions, ranging from 22 ± 2 °C. The scattering angle extended from 0° to 28°.

## 3. Results

### 3.1. Curing Conditions

**Figure 4:**
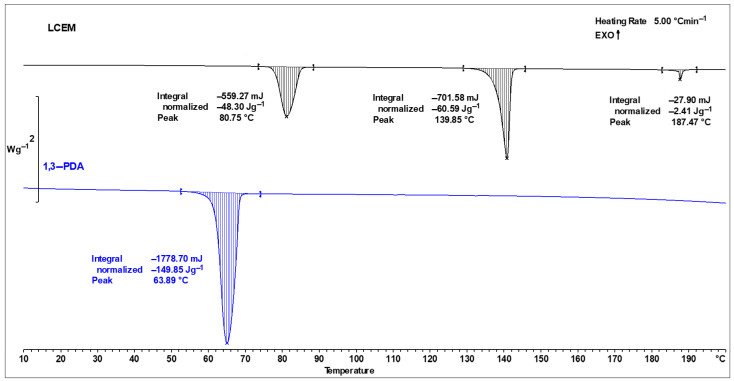
DSC curves presenting the phase transitions of individual components LCEM (black line) and 1,3-PDA (blue line).

During DSC analysis of the individual components of the mixture, the peaks characteristic of the liquid crystalline monomer and the aromatic diamine were recorded. The course of the DSC thermal curve obtained for the LCEM resin during the first measurement cycle (Figure 4, black line) suggests that the compound undergoes three endothermic transformations during heating. The first peak was observed at 81 °C and is a response to the polymorphic transformation of the compound. It indicates the moment when the LCEM monomer starts to have a different crystal structure. The next transition was recorded at about 140 °C when LCEM became a nematic liquid crystal. The last peak with the minimum at 187 °C shows the beginning of isotropization. During the heating of 1,3-phenylenediamine, the endothermic transformation above 64 °C was recorded in the thermogram (Figure 4, blue line), which was associated with the melting of this compound. All minimums appear at temperatures close to those given in the literature [13,21]. Table 2 summarizes the phase transformation parameters determined for both the monomer LCEM and 1,3-PDA amines.

**Figure 5:**
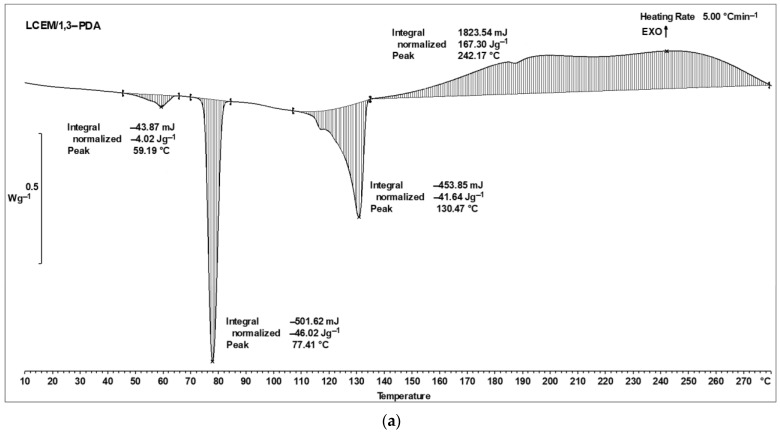

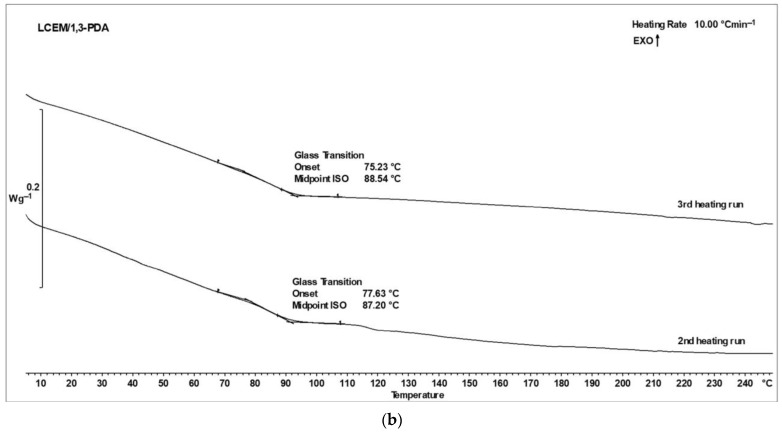
DSC curves of the LCEM/1,3-PDA composition: (**a**) 1st measurement cycle—heating to 280 °C with rate: 5 °C min^—1^ and (**b**) 2nd and 3rd measurement cycle; heating rate: 10 °C min^—1^.

The DSC analysis for the composition of LCEM/1,3-PDA (Figure 5a) showed interesting changes in the peaks appearing in the thermogram compared to the results that occurred for single compounds (Figure 4). It can be seen that the peak for the melting of aromatic diamine, which is close to 64 °C, is visible in the composition thermogram around 59 °C. This change may be induced by overlapping signals from 1,3-PDA and LCEM. The peak representing the polymorphic transformation of the liquid crystal monomer at 80 °C (Figure 4) is observed to occur at a slightly lower temperature in the case of the composition’s thermogram (around 77 °C). A similar situation appears for the liquid crystalline monomer, where the minimum peak for the monomer itself is assigned to a higher temperature (around 140 °C) than in the composite with a diamine (around 130 °C). The mixture begins to cross-link clearly around 150 °C and takes place with the release of energy. This results in a long exothermic peak that lasts until the end of the analysis.

The presence of that dual exothermic peak in the DSC thermogram is linked to a two-step cross-linking process, which happens to occur in liquid crystal materials [22]. It is associated with the orderliness change of the diamine during cross-linking. The functional groups of 1,3-PDA are primary. During cross-linking, they initially lose one hydrogen by joining the LCEM molecule, which appears in the thermogram in the form of the first exothermic maximum (~150–220 °C). They thus become secondary amines, and the continuation of the cross-linking reaction causes the amine group to transform into a tertiary one. This process shows up in the thermogram as the second maximum of this double peak above about 220 °C [23].

The cross-linking reaction described by a distinct and broad exothermic effect occurs right before the endothermic peak associated with melting the LCEM into the liquid-crystalline nematic phase. Due to their mutual interactions, the temperature peaks observed in mixed composition are slightly shifted in comparison to those of pure LCEM and the curing agent. The shape, enthalpy, and temperature of the endothermic peak that occurs around 130 °C suggest that changes in this temperature range are not solely related to the phase transition of the monomer. Simultaneously, around 130 °C, there is a partial exothermic cross-linking reaction occurring along with the transition of the LCEM. To confirm this hypothesis, TOPEM^®^ analysis was performed, in which the thermal effects were also analyzed using the DSC method.

Subsequently, during the overheating of the sample, the glass transition temperature (T_g_) of the composition was established according to the same standard as in Section 2.3. The phase transition temperatures are very similar in the second and third heating cycles (Figure 5a). The fact that they had almost identical values of 87 °C and 88 °C, respectively, and the shape of the curves, prove that the sample was completely cured during heating in the first cycle up to 280 °C (Figure 5a). The relatively low glass transition temperature can be attributed to the presence of long aliphatic chains in the LCER molecule, which give elasticizing properties [24].

#### 3.1.1. DSC-TOPEM^®^

DSC-TOPEM^®^ allows one to analyze the dynamic behavior of the sample over a wide frequency range in a single measurement. The reversing heat flow signal based on the quasi-static heat capacity and the non-reversing heat flow signals is a direct result of the correlation analysis [17].

**Figure 6:**
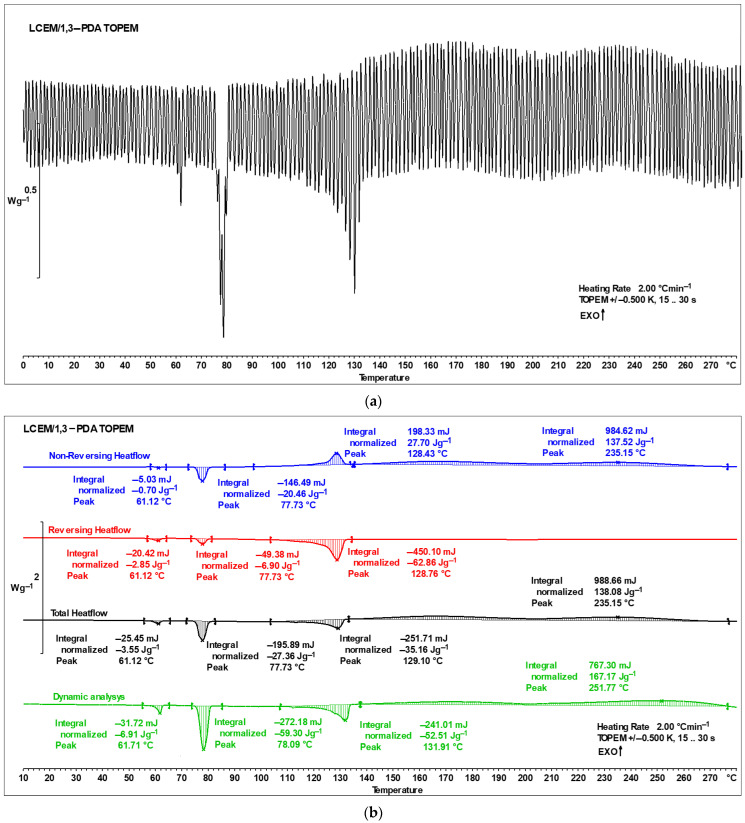
DSC-TOPEM^®^ curve of the LCEM/1,3-PDA: (**a**) before mathematical processing and (**b**) after mathematical processing.

The curve obtained after the DSC-TOPEM^®^ analysis (Figure 6a) requires mathematical processing, which allows for the proper interpretation of the results (Figure 6b). Temperature modulation in the DSC-TOPEM^®^ method gives the opportunity to elaborate on the results shown in Figure 6b, separating reversible effects (red curve) from irreversible effects (blue curve). As in the case of classic DSC analysis, peaks corresponding to individual thermal transitions of the mixture appear on the thermograms. Small minima at a temperature of around 61 °C illustrate the melting of diamine, while endothermic peaks on each of the curves around 78 °C are responsible for the polymorphic transformation of LCEM. Another change in the thermograms can be observed with peaks occurring around 130 °C. The irreversible exotherm process in the range of 110–135 °C suggests that the cross-linking reaction starts at a lower temperature than can be proven by the classical DSC analysis or the total heat flow (black curve) obtained from temperature-modulated DSC. It was decided to additionally employ the ^1^H-NMR method for the analysis, which involved examining a sample that had been heated to 134 °C.

**Figure 7:**
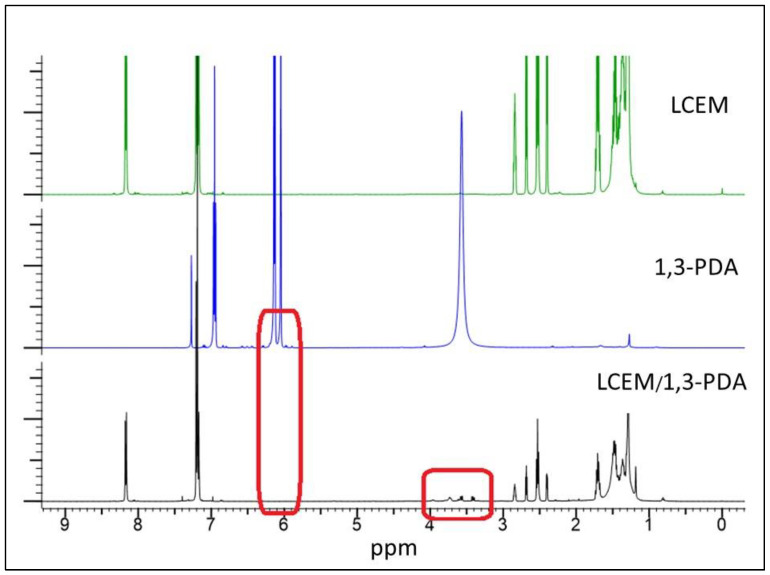
^1^H-NMR spectra of pure components before heating, and the LCEM/1,3-PDA mixture after heating to 134 °C. The red frame indicates the location where the signals for individual components of the mixture disappear.

Notable changes are observed in the resonance signals, including the appearance of additional signals and the disappearance of some (Figure 7). These alterations provide insight into the progression of the cross-linking reaction, which involves the opening of the epoxy ring and the formation of secondary amine groups. Within the 6–6.5 ppm range, there is an absence of resonance signals corresponding to 1,3-PDA. These signals represent protons in close proximity to amine groups, and their disappearance signifies a change in their magnetic environment, proving that the diamine react partially and the primary NH_2_ groups disappear. Further changes are evident in the 3–4 ppm range, where the symmetric singlet signal from NH_2_ protons splits. This indicates the formation of new amine groups with similar magnetic properties resulting from a reaction in which one proton interacts with the epoxy group of the liquid crystal monomer.

#### 3.1.2. Isothermal Analysis

**Figure 8:**
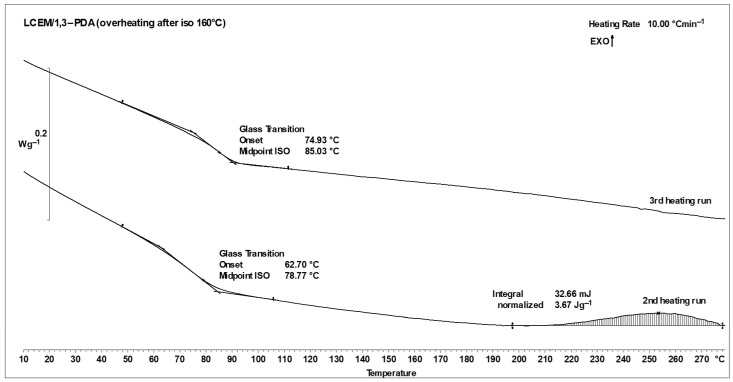
DSC curves of the LCEM/1,3-PDA composition heated isothermally in the first cycle to 160 °C—II and III measurement cycle; heating rate: 10 °C min^−1^.

The isothermal analyses performed for the samples as described in the Section 2 were aimed at establishing appropriate cross-linking conditions for the LCEM/1,3-PDA mixture. Samples tested at temperatures of 160 °C and 170 °C did not cross-link completely within 180 min during isothermal heating. As a result, when these samples were heated twice to a temperature of 280 °C, the end of one of the curves obtained in the thermogram was directed upward, which indicates the continuation of the cross-linking process. Moreover, the glass transition temperatures for the second and third heating processes are not very similar, which further confirms the assumption that the sample is not completely cross-linked. Figure 8 shows the results of overheating for the sample analyzed at 160 °C for 3 h.

**Figure 9:**
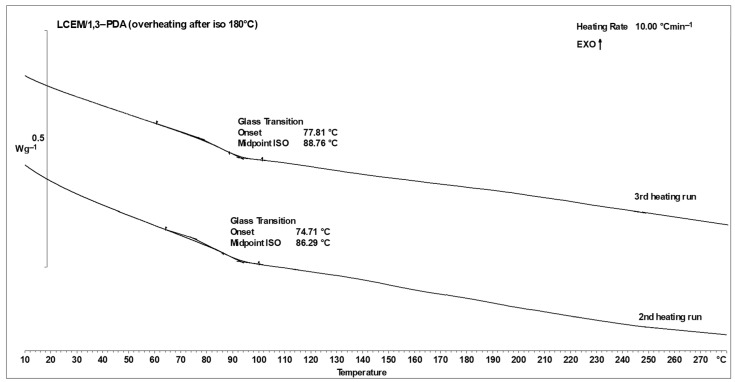
DSC curves of the LCEM/1,3-PDA composition heated isothermally in the first cycle to 180 °C—II and III measurement cycle; heating rate: 10 °C min^−1^.

Samples of the LCEM/1,3-PDA composition of LCEM/1,3-PDA analyzed at higher temperatures (180 °C, 190 °C, and 200 °C) for 3 h were completely cross-linked during isothermal analysis. During their overheating, no increase in the curves was recorded. The glass transition temperatures are very close to each other, which also proves complete the cross-linking of the sample. Figure 9 shows the results of overheating for the sample analyzed at 180 °C for 3 h. Similar results were obtained for two-stage cross-linked samples.

### 3.2. WAXS/SAXS Analysis

X-ray analyses of LCEM/1,3-PDA mixtures heated in isothermal conditions for 180 min were also performed, according to the DSC isothermal temperature program (Table 1). The results of the analysis showed that the cured compositions reveal some order under particular tested conditions. They were discussed on the basis of published studies by other researchers [25,26,27].

**Figure 10:**
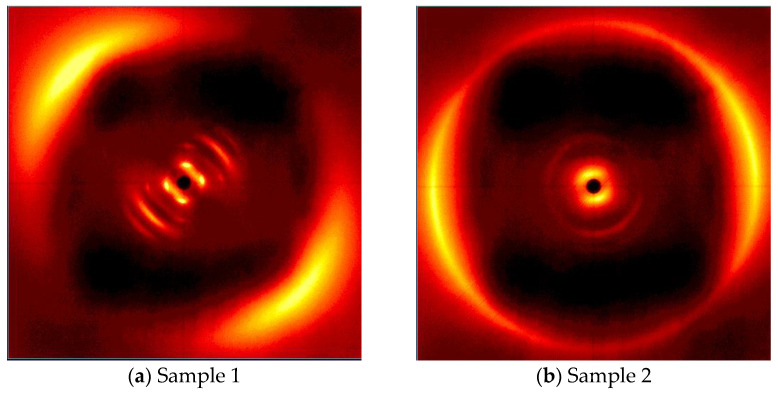
X-ray diffractograms of the LCEM/1,3-PDA composition cured at 160 °C for 180 min: (**a**) in a magnetic field of 1.2 T induction (Sample 1) and (**b**) without the presence of a magnetic field (Sample 2).

Figure 10 presents the results of the X-ray analysis of the LCEM/1,3-PDA composition cured at 160 °C for 180 min. In the case of Sample 1, the results of the WAXS analysis clearly indicate an order in the composition. Two pairs of arc reflections prove that the mesogenic parts of molecules are arranged in one plane. The fact that these reflections are set at different scattering angles may indicate the existence of a higher type of order in the cross-linked structure, such as smectic C [26]. In the case of Sample 2, the aforementioned arcs are less intense. However, the result of the analysis suggests that even without the presence of a magnetic field, the composition after cross-linking under such temperature conditions shows some degree of order, although to a lesser extent than Sample 1.

**Figure 11:**
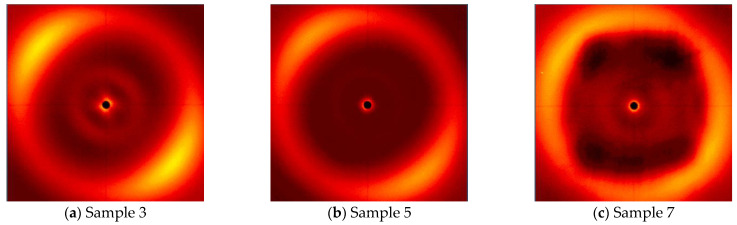
WAXS-2D of the LCEM/1,3-PDA composition cured in a magnetic field of 1.2 T induction, at: (**a**) 180 °C for 180 min (Sample 3), (**b**) 190 °C for 180 min (Sample 5), and (**c**) 200 °C for 180 min (Sample 7).

Subsequent X-ray analyses showed a decrease in order with an increasing sample curing temperature. Figure 11 shows the X-ray diagrams of the cross-linked LCEM/1,3-PDA mixture at 180 °C (Figure 11a), 190 °C (Figure 11b), and 200 °C (Figure 11c) for 3 h, in a magnetic field. The process carried out at 180 °C shows that the order of the composition occurs but to a lesser extent than at 160 °C. Similar signals are also visible for Sample 5 cured at 190 °C; however, they are much weaker than for previous samples. In the case of Sample 7 cross-linked at 200 °C, the ordering of mesogens is barely visible.

**Figure 12:**
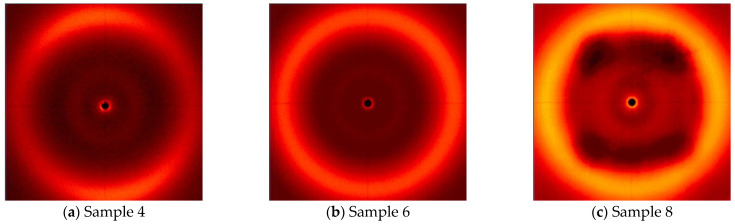
WAXS-2D of the LCEM/1,3-PDA composition cured without magnetic field at: (**a**) 180 °C for 180 min (Sample 4), (**b**) 190 °C for 180 min (Sample 6), and (**c**) 200 °C for 180 min (Sample 8).

Figure 12 shows samples cross-linked at analogous temperatures but without the presence of a magnetic field. In the case of Sample 4 (Figure 12a), a subtle thickening appears on the ring, similar to the sample cured at 160 °C, which means that the mesogens are partially ordered, even without the presence of a magnetic field. For Samples 6 (Figure 12b) and 8 (Figure 12c), there is no visible ordering according to the X-ray analysis.

**Figure 13:**
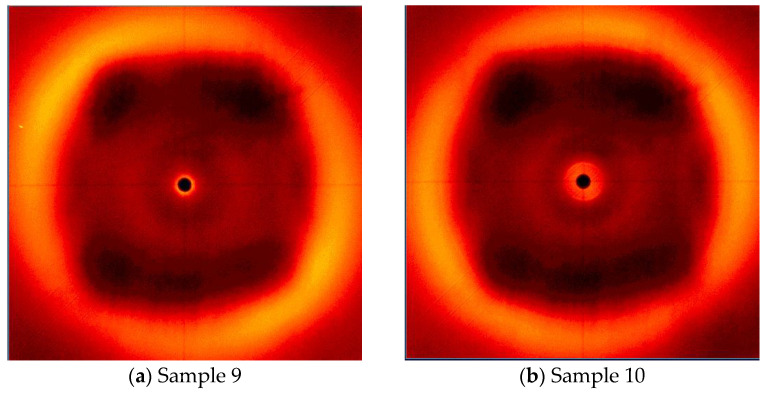

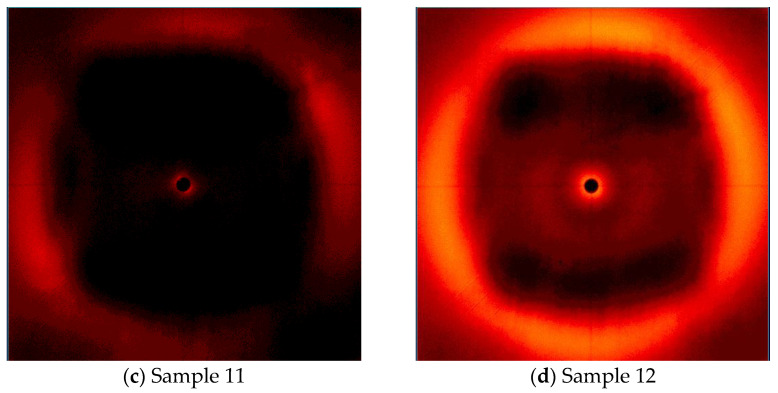
WAXS-2D of the LCEM/1,3-PDA composition cured at: (**a**) 160 °C/180 min + 200 °C/60 min in a magnetic field (Sample 9), (**b**) 160 °C/180 min + 200 °C/60 min without magnetic field (Sample 10), (**c**) 170 °C/180 min + 200 °C/60 min in a magnetic field (Sample 11), and (**d**) 170 °C/180 min + 200 °C/60 min without magnetic field (Sample 12).

Due to the interesting results obtained for Samples 1 and 2, it was decided to also test the samples cross-linked in two steps. This process consisted of keeping the sample at a constant temperature of 160 °C or 170 °C for 180 min and then increasing the temperature to 200 °C for 60 min. Similarly to the previous measurements, curing took place in the presence (Samples 9 and 11) and without the presence of a magnetic field (Samples 10 and 12). However, none of the samples examined during the X-ray analysis showed order in the structure (Figure 13).

Based on the results obtained from the isothermal analyses and the X-ray analyses, optimal cross-linking conditions for the composition were established. DSC analyses show that the samples analyzed at temperatures of 160 °C and 170 °C were not completely cross-linked. Therefore, appropriate conditions for cross-linking the sample involve the use of a temperature of at least 180 °C, or cross-linking the sample in two stages. In the case of X-ray analyses, the ordering of mesogens was recorded for samples cross-linked in the presence of a magnetic field at temperatures of 160 °C, 180 °C, and 190 °C. In the absence of a magnetic field or applying higher temperatures, as well as in the case of two-stage cross-linking, the order disappeared. Taking into account the results of all of these analyses, it was found that the most optimal conditions for cross-linking the LCEM/1,3-PDA composition are 180 °C for 180 min in the presence of a directing magnetic field. Under these conditions, the resulting sample exhibits both effective cross-linking and a well-ordered structure.

## 4. Discussion

The study focused on the characterization of cross-linking parameters and structural changes in a liquid-crystalline epoxy monomer system with an aromatic diamine curing agent. With techniques such as differential scanning calorimetry (DSC-TOPEM) and wide-angle X-ray scattering (WAXS) analysis, valuable insights regarding the material’s curing behavior and molecular arrangement were obtained. The DCS-TOPEM analysis helped to understand the underlying changes in the phase transition of the LCEM/1,3-PDA composition. WAXS analysis proved that, in general, an increase in the cross-linking temperature of the samples resulted in a loss of order in the composition. The presence of the magnetic field favored the arrangement of the mesogenic groups in the LCEM/1,3-PDA mixture. It was observed that the presence of that force field resulted in enhanced long-range ordering and improved intermolecular interactions, which are crucial factors influencing the mechanical properties of the liquid crystalline epoxy system. The cure characteristics determined by DSC and the structural changes revealed by WAXS analysis can help to understand the nature of these types of materials and guide the design and development of advanced liquid-crystalline, anisotropic epoxy thermosets with specific and defined morphologies, and properties tailored to various applications.

These findings contribute to our understanding of the fundamental properties and behavior of materials, paving the way for studies in various fields, from advanced materials engineering to nanotechnology. By unraveling the complexities of LCEM science, we can explore avenues for transformative applications and underscore its indispensable role in shaping the future of technology and innovation.

## Figures and Tables

**Table 1:** The samples and their curing conditions.

Sample Name	Curing Temperature/Time of Heating/°C/min	Magnetic Field Induction/T
Sample 1	160 °C/180 min	1.2 T
Sample 2	160 °C/180 min	0 T
Sample 3	180 °C/180 min	1.2 T
Sample 4	180 °C/180 min	0 T
Sample 5	190 °C/180 min	1.2 T
Sample 6	190 °C/180 min	0 T
Sample 7	200 °C/180 min	1.2 T
Sample 8	200 °C/180 min	0 T
Sample 9	160 °C/180 min + 200 °C/60 min	1.2 T
Sample 10	160 °C/180 min + 200 °C/60 min	0 T
Sample 11	170 °C/180 min + 200 °C/60 min	1.2 T
Sample 12	170 °C/180 min + 200 °C/60 min	0 T

**Table 2:** Phase transformation parameters of monomer LCEM and 1,3-PDA amine.

Sample	Phase Transformation Parameters Temperature/°C/Enthalpy/kJ g^−1^
LCEM	Cr_1_ (80.8 °C/−48.23 kJ g^−1^) Cr_2_ (139.9 °C/−60.75 kJ g^−1^) N (187.5 °C/−2.41 kJ g^−1^) I
1,3-PDA	T_m_ (63.9 °C/−149.4 kJ g^−1^)

Cr—crystal phase; N—nematic phase; I—isotropic phase; T_m_—melting temperature.

## Data Availability

Data are contained within the article.
